# Editorial: Intraoperative radiotherapy for gastrointestinal malignancy: updated evidence

**DOI:** 10.3389/fonc.2023.1217402

**Published:** 2023-05-15

**Authors:** Tadahiko Masaki, Felipe A. Calvo

**Affiliations:** ^1^ Department of Surgery, Kyorin University, Tokyo, Japan; ^2^ Department of Oncology, Clínica Universidad de Navarra, School of Medicine, Complutense University, Madrid, Spain

**Keywords:** intraoperative radiotherapy, gastrointestinal cancer, energy source, proton beam, neutron beam, electron beam

For the last several decades, intraoperative radiotherapy (IORT) has been used in either adjuvant or palliative settings for various types of neoplasms such as pancreas, breast, colorectal, gastric, head and neck, genitourinary, gynecological cancers, and retroperitoneal soft tissue sarcomas. In most cases, local control can be obtained by IORT with or without external beam radiotherapy (EBRT). Although much evidence has been accumulated in breast cancer from high quality of randomized controlled trials (RCT), the number of RCTs or meta-analyses are still limited in gastrointestinal (GI) cancers, and quality of evidence is not satisfactory.

In this Research Topic, we aimed to accumulate recent clinical and basic findings to improve efficacy of IORT in the treatment of GI cancers. There may be at least two aspects to improve efficacy of radiotherapy, such as tumor-related factors and treatment-related factors. Tumor-related factors include tumor size, histology, grade, location, primary or recurrent in nature, stage, and so on. Most of previous studies focused on these tumor-related factors.

Treatment-related factors include modality used, timing, route, dose, frequency, and energy source of radiation.

In spite of recent COVID-19 epidemic, we are lucky to collect 4 distinguished papers submitted to Frontiers in Oncology. All these 4 studies focused on treatment-related factors to improve efficacy of IORT.

Wang et al.’s paper was based on very unique basic research, which focused on improving efficacy of boron neutron capture therapy (BNCT). They constructed a tyrosine kinase inhibitor-L-p-boronophenylalanine (TKI-BPA) molecule, and tested its utility using gastric and pancreatic cancer cells. Future clinical application for GI cancers is awaited.

The other 3 papers were clinical studies for esophageal, rectal, and nasopharyngeal carcinoma, respectively. In patients with esophageal carcinoma, Suh et al. compared oncological outcomes and toxicities between photon and proton beam therapy, and showed overall and progression-free survivals, and radiotherapy-related toxicities were not significantly different between the two groups. In patients with low-lying T2 or T3 rectal adenocarcinomas undergoing concurrent chemo-EBRT combined with californium-252 neutron intracavitary brachytherapy (ICBT), Xiong et al. examined the efficacy of peritumoral injection of Amifostine. They showed that Amifostine pretreatment reduced toxicities of ICBT.

Finally, in patients with locally recurrent nasopharyngeal carcinoma, Lin et al. evaluated the oncological outcomes and late toxicities of three-dimentional high-dose-rate brachytherapy (3D-HDR-BT). They showed that, in the long term, local recurrence rate was lower in 3D-HDR-BT following EBRT than 3D-HDR-BT alone group.

Thanks to these valuable contribution to our Research Topic, the readers of our journal can understand that lots of researchers in the world are continuing their tremendous effort to improve clinical efficacy of electron, proton, or neutron beam radiotherapy. More scientific evidence will be obtained in the next 10 years’ time.

## Author contributions

All authors listed have made a substantial, direct, and intellectual contribution to the work and approved it for publication.

